# Proliferation- and apoptosis-associated factors in advanced prostatic carcinomas before and after androgen deprivation therapy: prognostic significance of p21/WAF1/CIP1 expression

**DOI:** 10.1038/sj.bjc.6690390

**Published:** 1999-05-01

**Authors:** G B Baretton, U Klenk, J Diebold, N Schmeller, U Löhrs

**Affiliations:** Institute of Pathology, Ludwig-Maximilians-University, Thalkirchnerstr. 36, D-80337 Munich, Germany; and Department of Urology, Klinikum Groβhadern, Ludwig-Maximilians-University, Marchioninistr. 15, D-81377 Munich, Germany

**Keywords:** prostate cancer pathology, androgen-independence, proliferation, apoptosis, p21/WAF1/CIP1 immunohistochemistry, prognosis

## Abstract

The molecular mechanisms leading to androgen-independent growth in prostate cancer (PC) are poorly understood. Androgen deprivation therapy (ADT) results physiologically in a decrease in proliferation and an increase in programmed cell death (PCD)/apoptosis. The aim of our study was to get more insight into these processes in prostatic carcinomas before and after ADT. For this purpose, immunohistologic staining for the androgen receptor (AR) molecule, the Ki-67 antigen, the *bcl-2* oncoprotein, the p53 protein and its physiologic effector, p21/WAF1, was performed on archival material. PCD was visualized by enzymatic detection of DNA fragmentation. Specimens from 69 PC patients after ADT were studied in correlation to histopathology and prognosis. In 42 cases, corresponding tumour tissue from the untreated primary tumours could be analysed comparatively. Before ADT, histologic grade was associated with Ki-67 index (*P* < 0.0001, Spearman correlation) and PCD rate (*P* < 0.05, Spearman correlation). Ki-67 index correlated with PCD rate (*P* < 0.05, Spearman correlation) and p21/WAF1 expression (*P* < 0.01, Fisher's exact test). p21/WAF1 expression was the only statistically significant prognostic factor for shorter survival (*P* < 0.002, log-rank test). All p21/WAF1-positive cases showed high Ki-67 index and high histologic grade. After ADT, loss of AR expression was associated with high Ki-67 index, whereas histologic signs of regression correlated negatively with Ki-67 index (*P* < 0.001, Pearson χ^2^ test). p21/WAF1 expression increased significantly (*P* < 0.02, McNemar test) and correlated with p53 accumulation (*P* < 0.0001, Pearson χ^2^ test). Most significant prognostic parameter after conventional ADT was high-rate p21/WAF1 expression (> 50% of tumour cells; *P* < 0.00001, log-rank test). This study demonstrates that p21/WAF1 overexpression before and after ADT characterizes a subgroup of advanced PC with paradoxically high proliferation rate and significantly worse clinical outcome. This finding might be clinically useful for planning therapy in these patients. © 1999 Cancer Research Campaign


British Journal of Cancer (1999) **80**(3/4), 546–555
© 1999 Cancer Research Campaign
Article no. bjoc.1998.0390

### Proliferation- and apoptosis-associated factors in advanced prostatic carcinomas before and after androgen deprivation therapy: prognostic significance of p21/WAF1/CIP1 expression

**GB Baretton[1], U Klenk[1], J Diebold[1], N Schmeller[2] and U Löhrs[1]**

[1]Institute of Pathology, Ludwig-Maximilians-University, Thalkirchnerstr. 36, D-80337 Munich, Germany, and [2]Department of Urology, Klinikum Groβhadern, Ludwig-Maximilians-University, Marchioninistr. 15, D-81377 Munich, Germany

**Summary** The molecular mechanisms leading to androgen-independent growth in prostate cancer (PC) are poorly understood. Androgen deprivation therapy (ADT) results physiologically in a decrease in proliferation and an increase in programmed cell death (PCD)/apoptosis. The aim of our study was to get more insight into these processes in prostatic carcinomas before and after ADT. For this purpose, immunohistologic staining for the androgen receptor (AR) molecule, the Ki-67 antigen, the *bcl-2* oncoprotein, the p53 protein and its physiologic effector, p21/WAF1, was performed on archival material. PCD was visualized by enzymatic detection of DNA fragmentation. Specimens from 69 PC patients after ADT were studied in correlation to histopathology and prognosis. In 42 cases, corresponding tumour tissue from the untreated primary tumours could be analysed comparatively. Before ADT, histologic grade was associated with Ki-67 index ($P$ < 0.0001, Spearman correlation) and PCD rate ($P$ < 0.05, Spearman correlation). Ki-67 index correlated with PCD rate ($P$ < 0.05, Spearman correlation) and p21/WAF1 expression ($P$ < 0.01, Fisher's exact test). p21/WAF1 expression was the only statistically significant prognostic factor for shorter survival ($P$ < 0.002, log-rank test). All p21/WAF1-positive cases showed high Ki-67 index and high histologic grade. After ADT, loss of AR expression was associated with high Ki-67 index, whereas histologic signs of regression correlated negatively with Ki-67 index ($P$ < 0.001, Pearson $\chi^2$ test). p21/WAF1 expression increased significantly ($P$ < 0.02, McNemar test) and correlated with p53 accumulation ($P$ < 0.0001, Pearson $\chi^2$ test). Most significant prognostic parameter after conventional ADT was high-rate p21/WAF1 expression (> 50% of tumour cells; $P$ < 0.00001, log-rank test). This study demonstrates that p21/WAF1 overexpression before and after ADT characterizes a subgroup of advanced PC with paradoxically high proliferation rate and significantly worse clinical outcome. This finding might be clinically useful for planning therapy in these patients.

**Keywords**: prostate cancer pathology; androgen-independence; proliferation; apoptosis; p21/WAF1/CIP1 immunohistochemistry; prognosis

Prostate cancer (PC) is one of the most common malignancies in the Western hemisphere. In Germany, PC is the third most common cause of male cancer mortality (Hölzel et al, 1996). Androgens (testosterone and 5 α-dihydrotestosterone) are essential for development, growth, differentiation and maintenance of organ structure of the prostate. Physiologically, androgen deprivation leads to apoptosis (Isaacs et al, 1994). The action of the androgens is mediated by the intracellular androgen receptor (AR) molecule (Trapman and Brinkmann, 1996). Initially, the growth of prostatic carcinomas is androgen-dependent in most cases. In locally advanced and/or metastasized PC, where prostatectomy is not curative, androgen deprivation therapy (ADT) performed either by castration or by other forms of endocrine manipulation (anti-androgens, luteinizing-hormone-releasing hormone (LH-RH) analogues) has been used for palliative therapy for more than 5 decades (Huggins and Hodges, 1941). However, nearly all cases become refractory to androgens within a few years. The molecular changes underlying androgen-independence and tumour progression are as yet widely unknown.

Unlike the oestrogen and progesterone receptor status in breast cancer, the expression of the AR molecule in PC does not correlate with clinical response to ADT (Miyamoto et al, 1993; Trapman and Brinkmann, 1996). This finding can be explained partly by mutations (Klocker et al, 1994; Peterziel et al, 1995; Taplin et al, 1995; Suzuki et al, 1996) or amplifications of the AR gene after ADT (Visakorpi et al, 1995). However, these aberrations are not present uniformly in hormone-independent carcinomas. Consequently, other mechanisms must be involved in the evolution of androgen-independent tumour progression as well.

In analogy to the physiological situation, the analysis of factors regulating cell proliferation and apoptosis may be important in this context, not only for scientific reasons, but also from a clinical point of view. To the best of our knowledge, however, studies have been missing up to now that investigate changes of proliferation- and programmed cell death (PCD)-associated factors in PC after ADT over a longer period of time. In order to avoid a therapy-related bias, the cases were classified into groups with either conventional ADT (by orchiectomy and/or drugs) or ADT and

*Received 28 July 1998*
*Revised 26 October 1998*
*Accepted 4 November 1998*

*Correspondence to:* GB Baretton

**Table 1** Gleason scores of the prostatic carcinomas after ADT

| Gleason score | All PC after ADT | | PC after ADT pretreated only by conventional ADT | |
|---|---|---|---|---|
| 4 | 2 (1[a]) | Low-grade (*n* = 5) | 2 (1[a]) | Low-grade (*n* = 4) |
| 5 | 0 | | 0 | |
| 6 | 3 (1[a]) | | 2 | |
| 7 | 1 | | 1 | |
| 8 | 9 (1[b]) | High-grade (*n* = 64) | 6 (1[b]) | High-grade (*n* = 36) |
| 9 | 26 (1[c]) | | 14 (1[c]) | |
| 10 | 28 (1[a], 1[b], 1[c]) | | 15 (1[a], 1[c]) | |
| Total | 69 | | 40 | |

Gleason scores and material (if not mentioned: transurethral resection/TUR; [a]transrectal punch biopsy, [b]subtotal transabdominal resection, [c]pulmonary metastasis) of the prostatic carcinomas after ADT discriminated according to subsequent therapy; all tumours under study (*n* = 69; median 9, mean 8.97 ± 1.3) and cases pretreated only by conventional androgen deprivation (castration and/or drugs; *n* = 40; median 9, mean 8.8 ± 1.5). For statistical calculations a grade compression was performed with a cut-point between scores 6 and 7 (low-grade vs high-grade).

additional therapy regimens (radiation or chemotherapy). The proliferation-associated Ki-67 antigen, the p53 protein and its physiologic effector, the p21/WAF1 protein, both known to be involved in the regulation of cell cycle and PCD/apoptosis, the apoptosis-inhibiting *bcl2*-oncoprotein, and the AR molecule were determined immunohistochemically. Furthermore, the PCD rate was visualized in situ by enzymatic detection of DNA fragmentation and quantified. The prognostic impact of these parameters was tested before and after long-standing ADT.

### MATERIALS AND METHODS

### Materials and patients

Formalin-fixed and paraffin-embedded tumour tissue from a total of 69 patients with locally advanced or metastasized primary prostatic carcinomas was studied after ADT with and without additional therapy regimens. The mean duration of ADT was 40.6 months ± 39.3 (median 30 months; range 0.4–169.4 months). The mean age of the patients at time of diagnosis was 67.5 ± 8.8 years (range 46–83 years).

Tumours were graded according to Gleason and Mellinger (Gleason, 1966; Gleason and Mellinger, 1974). For statistical calculations, a grade compression was performed with a cut-point between scores 6 and 7, i.e. low-grade vs high-grade (Gleason, 1992; Table 1). In 42 cases, paired specimens from both the untreated primary tumour and the recurrence after ADT were available (mean duration of ADT 26.8 ± 29.1 months; median 16.5 months, range 0.4–105.8 months; Table 2).

In 26 of these cases, only a conventional ADT was performed (‘core-group’) either by orchiectomy (OE; *n* = 8), or by OE and drugs (*n* = 11) or only by drugs (*n* = 7). For the ADT by drugs flutamide, fosfestrol, or cyproteronacetate were used primarily; later the patients were also treated with leuprorelinacetate, buserelinacetate, or goserelinacetate.

In the remaining 16 cases, an additional radiation (*n* = 2) or chemotherapy (*n* = 9), or a form of therapy that was not thoroughly documented (*n* = 5), was applicated in the further course. These cases were studied separately in order to avoid a therapy-related bias. For chemotherapy estramustin, 4′-epirubicin, or cisplatin were used.

In addition, tumour tissue from 27 patients after ADT was studied without paired material from the primary tumour. Fourteen of these patients were pretreated only by conventional ADT (OE: *n* = 4; OE + drugs: n = 7; only drugs: n = 3). Thus, tumour tissue from a total of 40 patients after conventional ADT alone was available. In the remaining 13 cases, an additional radiation (*n* = 3), chemotherapy (*n* = 8), or therapy not further specified (*n* = 2) had been performed.

**Table 2** Gleason scores of the untreated primary prostatic carcinomas

| Gleason score | All PC before treatment | | PC treated only by conventional ADT in the further course | |
|---|---|---|---|---|
| 4 | 3 (2[a]) | Low-grade | 1 (1[a]) | Low-grade |
| 5 | 1 (1[a]) | (*n* = 11) | 1 (1[a]) | (*n* = 9) |
| 6 | 7 (4[a], 2[b]) | | 7 (4[a], 2[b]) | |
| 7 | 12 (5[a]) | | 10 (5[a]) | |
| 8 | 8 (5[a]) | High-grade | 3 (3[a]) | High-grade |
| 9 | 9 (1[a]) | (*n* = 31) | 3 | (*n* = 17) |
| 10 | 2 (1[a]) | | 1 | |
| Total | 42 | | 26 | |

Gleason scores and material (if not mentioned = transurethral resection/TUR; [a]transrectal punch biopsy, [b]subtotal transabdominal resection) of the untreated primary prostatic carcinomas discriminated by subsequent therapy; all tumours under study (*n* = 42; median 7, mean 7.3 ± 1.5) and cases treated only by conventional ADT in the further course (castration and/or drugs; *n* = 26; median 7, mean 7.0 ± 1.3). For statistical calculations a grade compression was performed with a cut-point between scores 6 and 7 (low-grade vs high-grade).

© Cancer Research Campaign 1999

The material comprised 83 transurethral resections (TUR), 22 transrectal punch biopsies, four subtotal transabdominal prostatic resections, and two pulmonary metastasis (Tables 1 and 2). All samples were routinely fixed in 4% buffered formalin and embedded in paraffin (total of 111 paraffin blocks).

In the tumour tissue after ADT nuclear size reduction, loss of recognizable nucleoli, chromatin condensation, nuclear pyknosis and vacuolization of the cytoplasm of the tumour cells were recorded as histopathological signs of regression (Civantos et al, 1995). If these features were lacking, the case was classified histopathologically as regression-negative.

In 66 cases, clinical follow-up data were available, including all patients with paired tumour samples before and after ADT (mean time of observation: 59.5 ± 48.9 months; median 47 months; range: 1–215 months). Twenty-seven of 66 patients died.

### Methods

### *Immunohistochemistry*

Sections (3–4 μm thick) were cut from the paraffin blocks and mounted on Super-Frost/Plus microscope slides (Menzel, Germany) and dried for 24 h at 56°C. After deparaffinization, rehydration and washing in phosphate-buffered saline (PBS) the slides were placed in a Coplin jar that was filled with 10 mM citrate buffer (pH 6.0). The jar was heated in an autoclave (Webeco, Germany) at 120°C (1.2 bar) for 20 min (Piffko et al, 1995). The jar was then allowed to cool at room temperature. After three washings in PBS the slides were incubated with 75 μl of the primary monoclonal antibodies prediluted in 1% bovine serum albumin (Sigma, Deisenhofen, Germany) in PBS for 20 h at 6°C in a wet chamber (Table 3).

The reactions were visualized by an amplified biotin–streptavidin method (Super Sensitive Multilink Immunodetection System™; BioGenex, San Ramon, CA, USA). For this purpose, after washing in PBS, the sections were incubated for 30 min (21°C, wet chamber) with the biotinylated anti-mouse antibody (Super Sensitiv Multilink, HK 340-9K; BioGenex, USA). After washing again in PBS an incubation with alkaline–phosphatase conjugated streptavidin (Super Sensitiv Label, HK 331-9K; BioGenex, USA) was performed (30 min, 21°C, wet chamber). Fast red–naphthole AS-MX phosphate (F-4523; Sigma, Germany) served as chromogene. Finally, the slides were counterstained with haematoxylin and mounted in Aquatex (Merck, Germany).

### *Analysis of the immunohistochemical reactions*

#### *Ki-67*

According to the immunoreaction in 1000 tumour cells/tumour the mean percentage of nuclei with a positive immunoreaction was determined (= Ki-67 index). A Ki-67 index was classified as high if it was ≥ 14.7%, which represented the median value in the 42 primary PC before ADT.

#### *AR*

A tumour was classified as positive for AR molecule expression if a clear positive nuclear reaction was present in > 95% of the tumour cells. Cases with no, or only single, positive cells (< 5%) were regarded as negative.

#### *p53 and p21/WAF*

The results of the p53 and p21/WAF1 immunoreaction were recorded as positive if a distinct nuclear staining was seen in at least 20% of the tumour cells in a microscope field at 100-fold magnification (Zeiss Axioskop microscope: objective 10/0.30, ocular 10×/20; Zeiss, Germany). A further subclassification was performed in cases with p21/WAF1 immunoreactivity in > 50% of all tumour cells; those cases were classified as high-rate positive. Cases with no, or only single, positive cells were regarded as negative.

This classification was used because there is increasing evidence that p53 immunoreactivity in only a few tumour cells, or even normal cells, does not correlate with a *TP53* mutation, but represents overexpression of wild-type p53 due to physiologic DNA repair. Accordingly, the effector of the wild-type p53-protein, i.e. p21/WAF1, can physiologically show a positive reaction in single cells.

#### *bcl-2*

The immunohistochemical results for *bcl-2* were recorded as positive if the tumour cells showed an unequivocally strong cytoplasmatic immunoreaction in at least 20% of the tumour cells in a microscope field at 100-fold magnification (see above). Cases with only equivocally faint staining were regarded as negative. Positive-reacting lymphocytes and basal cells in preserved non-neoplastic prostatic glands served as internal control.

### *Detection of apoptotic cells in paraffin sections*

DNA fragments of apoptotic cells were visualized by an enzymatic reaction (Gavrieli et al, 1992; Ansari et al, 1993). After dewaxing in xylene, rehydration, washing in PBS and pretreatment of the sections with proteinase K (Sigma, Germany; 40 μg $ml^{-1}$ in PBS, 21°C, 45 min), the endogenous peroxidase activity was blocked in 3% hydrogen peroxide for 5 min. Subsequently, the terminal transferase reaction was performed using the ApopTag™ kit (In Situ Apoptosis Detection Kit, Peroxidase, S 7100-Kit; ONCOR, Gaithersburg, MD, USA). DAB (3,3'-diaminobenzidintetrahydrochloride; D-51905; Sigma, Germany) served as chromogen (one tablet in 15 ml PBS). After 5 min of incubation with DAB,

**Table 3** List of primary antibodies used for immunohistochemistry

| Antibody | Clone | Company | Dilution[a] |
|---|---|---|---|
| Ki-67 | MIB 1 | Dianova, Hamburg, Germany | 1:1000 |
| p53 | DO 1 | Dianova, Hamburg, Germany | 1:100 |
| p21/WAF1 | clone EA10 | Dianova, Hamburg, Germany | 1:100 |
| bcl-2 | clone 124 | DAKO, Hamburg, Germany | 1:750 |
| Androgen receptor | F 39.4.1 | BioGenex, San Ramon, CA, USA | 1:10 |

Primary monoclonal antibodies used for the immunohistochemical reactions and dilutions (*in 1% bovine serum albumin (Sigma, Germany) in PBS).

© Cancer Research Campaign 1999

the slides were washed in running tap water for 10 min and in double-distilled water (DDW) for 5 min. Afterwards, the slides were counterstained with methyl green (HK 141-7K; BioGenex, USA) for 10 min under a cover-glass at 21°C, washed again in DDW and mounted with Aquatex (Merck, Germany).

As positive controls, preserved non-neoplastic prostatic glands within the slides from primary tumours, or lymphocytes in lymph follicles, were used. Since the enzymatic reaction described labels, both apoptotic cells and areas of necrosis, the only labelled cells that were regarded as positive were those that showed additional characteristics of apoptosis, e.g. isolated localization within an intact cell complex without an inflammatory reaction. At least 10 000 tumour cells per case were evaluated. The results were expressed as positive cells per 1000 tumour cells.

As 2.5% represented the median value of apoptotic cells in the 42 primary tumours before ADT, a PCD index < 2.5% was considered low, and a PCD index > 2.5% high.

### *Statistical analyses*

Mean parameter values ± standard error of the mean (S.E.M.) were statistically compared by Spearman correlation, $\chi^2$ analysis, Fisher's exact test, Wilcoxon's signed rank test, and the McNemar test (Hollander and Wolfe, 1973; Breslow and Day, 1980). Survival analyses were performed by Kaplan–Meier curves and the log-rank test using the SPSS statistical software (SPSS Inc., Chicago, IL, USA). $P < 0.05$ was regarded as statistically significant.

### RESULTS

### Prostatic carcinomas before ADT

#### *AR molecule*

All primary PC showed a nuclear expression of the AR molecule before ADT.

#### *bcl-2*

Twenty of 42 PC before ADT (48%) exhibited a positive cytoplasmatic immunohistochemical reaction for *bcl-2*. In the subgroup of 26 carcinomas treated later only by conventional ADT, 15 cases were *bcl-2*-positive (58%). The pattern of immunostaining was not uniform within the tumours; clusters of cells that reacted positively alternated with only faint staining areas within an individual carcinoma without correlation to histologic grade of the tumours or the other parameters under study. A positive cytoplasmatic *bcl-2* reaction was also present in the basal cells in non-neoplastic glands in the adjacent prostate tissue corresponding with the physiologic proliferative zone and in reactive lymphocytes/lymphoid follicles infiltrating the prostate.

#### *p53*

Eleven of 42 untreated carcinomas (26%) were immunohistochemically p53-positive (including six of the 26 cases (23%) with subsequent conventional ADT without additional radiation or chemotherapy), whereas in the remaining tumours no nuclear p53 accumulation could be observed. In all positive carcinomas, however, parts of the neoplasms remained negative. All p53-positive carcinomas were histologically high-grade tumours; however, the correlation between p53 and histological grade was not statistically significant. Furthermore, no correlations between p53 accumulation and the other parameters under study could be found. The preserved non-neoplastic prostatic tissue showed no p53 immunoreactivity.

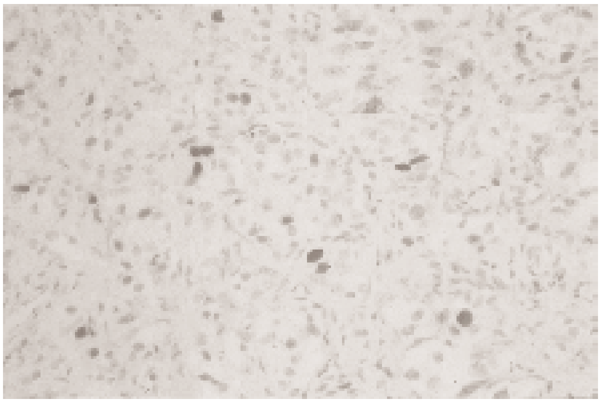

**Figure 1** Focally positive immunohistological reaction for p21/WAF1 (> 20% of tumour cell nuclei) in a prostatic adenocarcinoma (Gleason score 7) before androgen deprivation therapy (monoclonal antibody clone EA 10, haematoxylin, original magnification 200×)

#### *p21/WAF1*

Seven of 42 PC before ADT (17%) showed a positive nuclear immunoreaction for p21/WAF1. In this group, p21/WAF1 expression was correlated positively with Ki-67 index ($P < 0.01$, Fisher's exact test). In the subgroup of 26 patients with conventional ADT in the later course, four tumors were p21/WAF1-positive (15%). The reaction pattern was heterogeneous in the tumours demonstrating positive foci of tumour cells alternating with negative areas (Figure 1). The distribution of cases with focal and high-rate p21/WAF1 immunoreactivity is shown in Table 4. No statistically significant correlations could be established between p21/WAF1 immunoreactivity, histologic tumour grade or other parameters under study. p21/WAF1-positive tumours were histologically mostly high-grade carcinomas, showed high Ki-67 and PCD index, and were *bcl-2*-positive; however, these associations were statistically not significant. In the non-neoplastic prostate tissue, very few epithelial cells showed an occasional positive p21/WAF1 immunoreactivity (< 1%) in all cases.

**Table 4** p21/WAF1 immunoreactivity in prostatic carcinomas before and after ADT

| | p21/WAF1 immunoreactivity | | |
|---|---|---|---|
| | Focal positivity[a] *n* (%) | High-rate positivity[b] *n* (%) | Total pos. *n* (%) |
| *Before ADT* | | | |
| All cases ($n = 42$) | 6 (14) | 1 (2) | 7 (17) |
| 'Core-group' ($n = 26$) | 3 (12) | 1 (4) | 4 (15) |
| *After ADT* | | | |
| All cases ($n = 69$) | 21 (30) | 10 (15) | 31 (45) |
| Cases with paired samples ($n = 42$) | 12 (29) | 7 (17) | 19 (45) |
| 'Core-group' ($n = 26$) | 6 (23) | 6 (23) | 12 (46) |

p21/WAF1 immunoreactivity in prostatic carcinomas before and after ADT. Cases with [a]focal p21/WAF1 expression (> 20% of tumour cells in one microscope field, 100×), [b]high-rate p21/WAF1 immunoreactivity (> 50% of all tumour cells), and the total number of p21/WAF1-positive cases in the different subgroups under study.

© Cancer Research Campaign 1999 

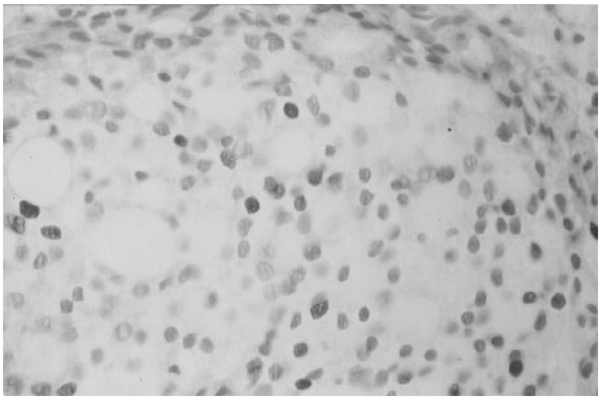

**Figure 2** Cribriform prostatic carcinoma (Gleason score 8) before androgen deprivation therapy with high PCD-/apoptotic rate (brown nuclei; PCD-index 5%; terminal transferase reaction, methyl green, original magnification 500×)

### *Ki-67*

The mean value of the Ki-67 index was 17.4 ± 12.1% in the untreated PC. According to the defined classes of proliferation, the cases with low and high Ki-67 were distributed equally (50%). In the core group, 62% of the cases ($n$ = 16) showed a low, and 38% ($n$ = 10) a high, Ki-67 index respectively. Statistically significant positive correlations could be established between Ki-67 index and histologic grade ($P < 0.0001$, Spearman correlation) as well as between Ki-67 index and PCD index ($P < 0.05$, Spearman correlation).

### *poptosis*

In the 42 tumours before ADT, the mean PCD index was 2.96 ± 2.6%. The PCD index was graded as low (< 2.5%) in 22 cases (52%) and as high in 20 carcinomas (48%; Figure 2). From the 26 prostate cancer patients treated subsequently only by conventional ADT, 17 tumours (65%) showed a low PCD index and nine carcinomas (35%) exhibited a high PCD index. The PCD index correlated statistically significantly with tumour grade ($P < 0.05$, Spearman correlation) and, as mentioned above, with Ki-67 index ($P < 0.05$, Spearman correlation). Moreover, a positive correlation between PCD index and patients, age could be found ($P < 0.02$). In nearly all cases, scarce apoptotic cells were also detected by terminal transferase reaction in the secretory epithelial cells of non-neoplastic prostatic glands.

### *Prognostic significance*

The only parameter with statistically significant prognostic impact on prognosis before ADT was the p21/WAF1 expression of the tumour cells. Patients with p21/WAF1-positive carcinomas showed a statistically significant shorter survival time than those with p21/WAF1-negative tumours ($P < 0.0002$, log-rank test, in all untreated prostatic carcinomas, Figure 3A; $P < 0.00001$, log-rank test, in the 'core-group' treated only with conventional ADT, Figure 3B).

### Prostatic carcinomas after ADT

### *Histologic grade and signs of tumour regression*

In the cases where paired tumour samples before and after ADT were available, the Gleason score increased significantly after therapy ($P < 0.001$, Wilcoxon's signed rank test).

From the total of 69 PC after ADT, 24 carcinomas (35%) showed histologic signs of tumour regression. In the subgroup treated only with conventional ADT, 18 of 40 cases (45%) demonstrated histologic changes that could be related to the therapy.

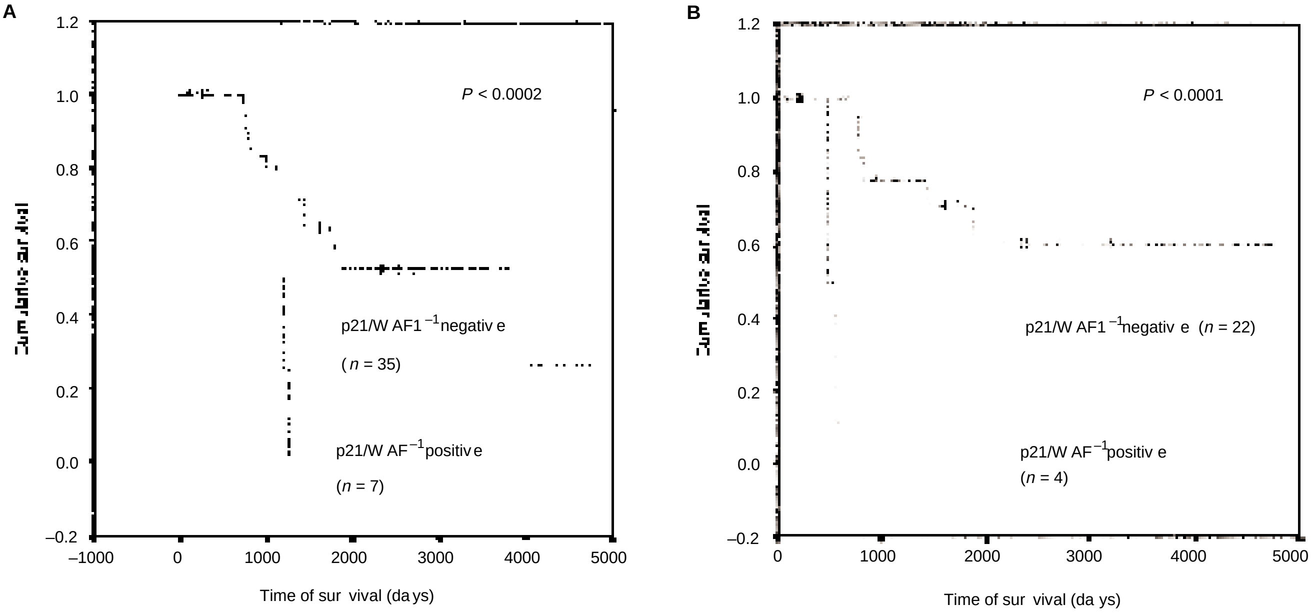

**Figure 3** Kaplan–Meier survival curves for patients with advanced prostatic carcinomas before androgen deprivation therapy. Statistically shorter survival for patients with p21/WAF1 expression of the tumour cells (> 20% in a microscope field at 100-fold magnification) in the untreated primary tumours. (**A**) In all patients with prostatic carcinomas under study ($n$ = 42; $P < 0.002$; log-rank test). (**B**) In the subgroup treated by conventional ADT only in the further course ($n$ = 26; $P < 0.00001$; log-rank test)

 © Cancer Research Campaign 1999

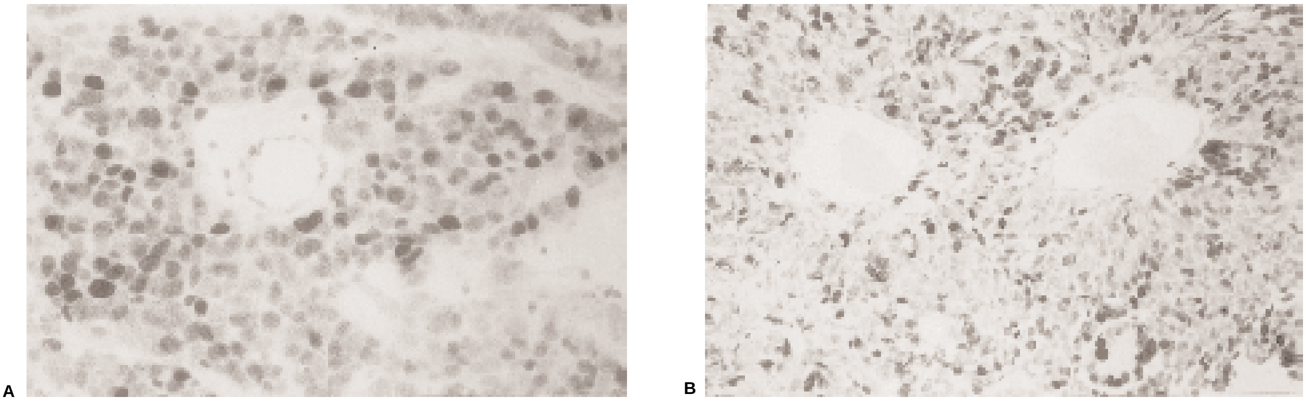

**Figure 4** (**A**) Immunohistological high-rate nuclear expression of p21/WAF1 (> 50% of tumour cell nuclei) in a poorly differentiated prostatic carcinoma (Gleason score 10) after androgen deprivation therapy (monoclonal antibody clone EA 10, haematoxylin, original magnification 250×). (**B**) Serial section of the same tumour showing a high Ki-67 labelling index (40%) in the same area (monoclonal antibody MIB1, haematoxylin, original magnification 200×)

Independently, whether only a conventional ADT or an additional radiation or chemotherapy had been performed, in all of these cases, the histologic signs of regression were only slight and could be observed only in a part of the tumour tissue.

### *AR molecule*

After ADT, 61 PC (88%) expressed the AR molecule. After pretreatment with conventional ADT only, 36 of 40 carcinomas (90%) were AR-positive, including all of the 26 cases in which also paired tumour tissue before ADT had also been analysed. The remaining cases showed no immunoreactivity or a positive reaction only in a few cells (< 5%). The loss of the AR molecule was associated with high-rate p21/WAF1 ($P < 0.02$, Fisher's exact test) and high-rate *bcl-2* ($P < 0.04$, Fisher's exact test) and with a tendency to high Ki-67 index.

### *bcl-2*

In 42 of the carcinomas after ADT (61%), a positive cytoplasmatic immunoreactivity for *bcl-2* could be found. After conventional ADT, 58% of the tumours showed *bcl-2* positivity. Thus, a trend towards a higher rate of *bcl-2* expression after ADT could be registered.

### *p53*

In 26 PC (38%), a nuclear p53 accumulation could be observed. In the ADT subgroup without additional therapeutic regimens, 12 of 40 tumours (30%) reacted p53 positively; in the 26 core-group cases with paired tumour samples this rate was 39%. In comparison with the results before ADT the rate of aberrant immunohistochemical p53 expression increased insignificantly. After ADT, p53 accumulation and p21/WAF1 expression were correlated statistically significantly ($P < 0.0001$, Pearson $\chi^2$ test).

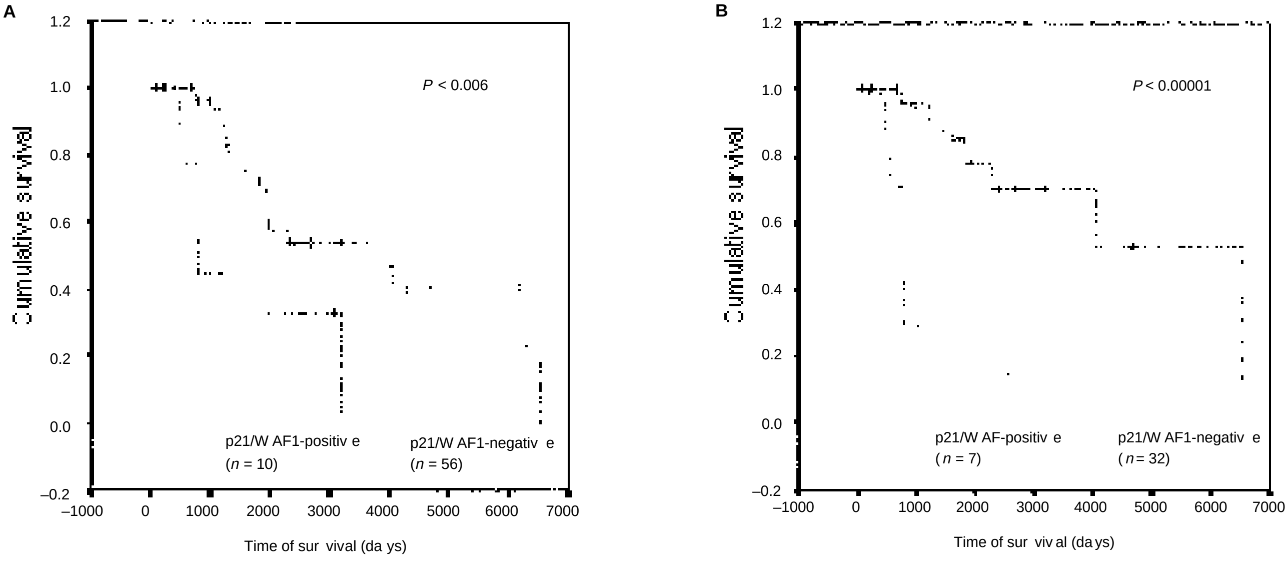

**Figure 5** Kaplan–Meier survival curves for patients with advanced prostatic carcinomas after androgen deprivation therapy. Statistically shorter survival for patients with high-rate p21/WAF1 expression of the tumour cells (> 50% of all tumour cells) in the recurrences. (**A**) In all patients with prostatic carcinomas under study ($n = 66$; $P < 0.006$; log-rank test). (**B**) In the subgroup pretreated by conventional ADT only in the further course ($n = 40$; $P < 0.00001$; log-rank test)

*© Cancer Research Campaign 1999*

**Table 5** Expression patterns of proliferation-/apoptosis-regulating factors, proliferative activity (Ki-67 index), and apoptosis/PCD rate in advanced prostatic carcinomas before and after ADT and prognostic significance

| Parameter | PC 'Core-group' (= paired specimens; *n* = 26) | | | | PC All tumours | | | | | |
|---|---|---|---|---|---|---|---|---|---|---|
| | Before ADT | Progn. sign. | After ADT only | Progn. sign. | All PC before ADT (*n* = 42) | Progn. sign. (*n* = 42) | All PC after ADT +/– additional therapy (*n* = 69) | Progn. sign. (*n* = 66) | PC after ADT only (*n* = 40) | Progn. sign. (*n* = 40) |
| AR expression | 100% | – | 92% | – | 100% | – | 88% | – | 90% | – |
| bcl-2 pos. | 58% | – | 58% | – | 48% | – | 61% | – | 58% | – |
| p53 pos. | 23% | – | 39% | – | 26% | – | 38% | – | 30% | – |
| p21/WAF1 pos. | 15% | $P < 0.00001$ | 46%[a] | $P < 0.002$ | 17% | $P < 0.0002$ | 45% | $P < 0.006$[b] | 18% | $P < 0.00001$[b] |
| Ki-67 index high | 38% | – | 54% | $P < 0.04$ | 50% | – | 64% | – | 63% | – |
| PCD rate high | 35% | – | 42% | $P < 0.03$ | 48% | – | 49% | – | 40% | – |

Expression patterns of proliferation-/apoptosis- regulating factors, proliferative activity (Ki-67 index), and apoptosis/PCD rate in advanced prostatic carcinomas before and after ADT and prognostic (progn. significance (sign.) of the parameters (log-rank test). Left: Cases with paired specimens from both the untreated primary tumour and the recurrence after conventional ADT only; [a]statistically significant increase after ADT ($P < 0.02$, McNemar test). Right: All cases under study before and after ADT with and without additional chemotherapy or radiation; [b]high rate p21/WAF1-overexpression only.

### *p21/WAF1*

Thirty-one PC analysed after ADT (45%) showed a positive nuclear reaction for p21/WAF1 (Figure 4A). The distribution of cases with focal and high-rate p21/WAF1 immunoreactivity is shown in Table 4. In comparison to the status before ADT, the rate of p21/WAF1 overexpression increased significantly ($P < 0.004$, McNemar test). In the paired tumour samples after conventional ADT only, 12 of 26 prostatic carcinomas reacted p21/WAF1 positively (46%; $P < 0.02$, McNemar test). As mentioned above, p21/WAF1 expression correlated with p53 accumulation ($P < 0.0001$, Pearson $\chi^2$ test), as well as with Ki-67 index ($P < 0.01$, Pearson $\chi^2$ test; Figure 4B).

### *Ki-67*

After ADT, the mean value of the Ki-67 index was 26.3 ± 18.5% in the PC. The Ki-67 index was classified as low in 25 cases (36%), and as high in 44 cases (64%). In the 40 patients treated only with conventional ADT, these rates were 38% and 62% respectively. In the 'core-group', the distribution of tumours was 46% low and 54% high Ki-67 index. A significant increase towards higher Ki-67 index was shown in paired sample analysis ($P < 0.01$, Wilcoxon's signed rank test). The Ki-67 index was statistically significantly correlated with grading ($P < 0.001$, Spearman correlation) and PCD rate ($P < 0.05$, Spearman correlation). Furthermore, in tumours with histologic signs of regression, the rate of cases with low Ki-67 index was statistically significantly higher (67%) than in the carcinomas without tumour regression (14%; $P < 0.01$, Pearson $\chi^2$ test).

### *Apoptosis*

The mean value of the PCD index in the 69 prostate carcinomas after ADT was 3.8 ± 3.6%. According to the limits mentioned above, the PCD index was classified as low in 35 cases (51%) and high in 34 cases (49%). In the subgroup treated only with conventional ADT, the cases with low and high PCD index were distributed into 60% low and 40% high PCD index. Thus, in comparison with the status before ADT, a slight decrease of cases with high PCD index could be found. PCD index correlated with grading ($P < 0.002$, Spearman correlation) and with Ki-67 index ($P < 0.05$, Spearman correlation).

### *Prognostic significance*

In the total of 66 patients with all forms of treatment and clinical follow-up data available, patients whose tumours showed high-rate p21/WAF1 had a significantly shorter survival time than the others ($P < 0.006$, log-rank test; Figure 5A). This result became even more evident in the subgroup treated by conventional ADT only ($n = 40$; $P < 0.00001$, log-rank test; Figure 5B). Moreover, in the 'core-group' pretreated with conventional ADT only, the Ki-67 index and the PCD rate were statistically significant prognostic parameters as well (Table 5).

### DISCUSSION

The molecular mechanisms underlying androgen-independent growth in PC after long standing ADT are still poorly understood. Elucidation of these processes might lead to improvement of existing endocrine therapeutic regimens or to the development of new forms of therapy (Trapman and Brinkmann, 1996).

In the present investigation, we analysed immunohistochemically several factors known to be involved in the regulation of proliferation and PCD/apoptosis. By comparing the results from both the primary untreated PC and the recurrences after a longer period of ADT from the same patients, we hoped to gain a better understanding of these pathways in PC tumour cells. Since in several cases other therapy regimens were performed in addition to conventional ADT, the collective was subclassified into groups with either conventional ADT alone or with ADT and additional chemotherapy or radiation to avoid therapy-related influences on the results. On the one hand, the discrepancies between these groups observed in our study concerning the correlations and/or the prognostic impact of the analysed parameters support the necessity of a stratification of the patients with advanced prostate cancer according to the applied therapy regimen (Table 5). On the

 © Cancer Research Campaign 1999

other hand, these findings may explain differences between the results of our study and other investigations published in the literature, where no stratification was performed.

In accordance with the majority of reports in the literature, the immunohistological AR expression was preserved in all tumours of our series before ADT and did not correlate with clinical and histopathologic features (Trapman and Brinkmann, 1996). After ADT, loss of the AR molecule could be found in less than 10% of cases. The observed association between AR loss and high Ki-67 index suggests that the AR-regulated growth control is overruled in this subset of cases by other factors, e.g. activation of 'classical' oncogenes and/or inactivation of tumour suppressor genes. This hypothesis is further supported by the observation that the Ki-67 index (and the PCD rate) were prognostically relevant for survival in the subgroup after conventional ADT.

Furthermore, our data show that the physiologic regulation of cell proliferation and apoptosis are obviously defective already before ADT in the majority of advanced PC. Before and after ADT the PCD rate was associated only with tumour grade and proliferative activity assessed by the Ki-67 index. No correlation could be observed between PCD rate and the PCD-inhibiting *bcl-2* oncoprotein expression (Korsmeyer, 1992*b*), supporting the view that regulation of apoptosis is complex and *bcl-2* is not the only factor involved in this process (Krajewska et al, 1996). For example, apoptosis may also be initiated by incompatible or conflicting growth signals (Stewart, 1994), e.g. by c-*myc*, or by the activation of a cyclin-dependent kinase at an inappropriate time during the cell cycle (Evan et al, 1992). In agreement with earlier reports, the rate of tumours expressing *bcl-2* was higher after ADT (McDonnell et al, 1992; Colombel et al, 1993; Westin et al, 1995), although the increase was not statistically significant in our cases. In contrast to other studies, no correlations between *bcl-2* overexpression and PCD rate, p53 accumulation, or clinical outcome could be observed before and after ADT. In these investigations, however, either locally confined, i.e. resectable prostatic carcinomas (Bubendorf et al, 1996), or short-term changes after ADT were studied (McDonnell et al, 1992; Westin et al, 1995).

The *TP53* tumour suppressor gene is the most commonly mutated gene in human cancers (Levine et al, 1991). The p53 protein plays a central role in the regulation of proliferation and PCD. Its main physiologic function is controlling the integrity of the DNA (Lane, 1992). If DNA damage is present the cell cycle will be stopped in the G1 phase mediated by the p53 effector protein p21/WAF1, and DNA repair is induced. If the damage is irreparable, apoptotic cell death is induced. Thus, inactivation of p53 is able to block apoptotic cell death (Korsmeyer, 1992*a*). As functionally relevant mutations of the *TP53* gene mostly result in an abnormal stabilization of the mutated protein, its accumulation can be visualized by immunohistochemistry, whereas the wild-type p53 escapes detection due to the short half-life time. In PC, p53 immunohistology was proven to correlate well with *TP53* gene mutations (Wertz et al, 1996). Nonetheless, *TP53* mutations seem to be a late event in PC progression and are identified mainly in poorly differentiated and metastatic tumours, indicating an association with poor prognosis (Visakorpi et al, 1992; Bookstein et al, 1993; Navone et al, 1993; Eastham et al, 1995; Kubota et al, 1995; Shurbaji et al, 1995) The rates of approximately 30% and 40% of p53 positivity in advanced PC before and after ADT, respectively, are within the range of results for abnormal p53 accumulation published in the literature. In our series, however, no statistically significant correlations between p53 accumulation and clinicopathological features or prognosis could be established.

Obviously, the main result of our study was that overexpression of the p21/WAF1 protein, the effector of the wild-type p53 protein, was statistically significantly correlated with a shorter survival time of the patients (Figures 3 and 5). As p21/WAF1 is physiologically a potent inhibitor of cyclin-dependent kinases (CDK) and can be regarded as a negative regulator of the cell cycle inducing either p53-mediated G1 arrest or apoptosis, this finding was unexpected (El-Deiry et al, 1994; Li et al, 1994; Polyak et al, 1997). Accordingly, loss of p21/WAF1 expression was described mainly in more aggressive tumour variants or advanced tumour stage (Ellis et al, 1997; Gomyo et al, 1997; Harada et al, 1997; Maelandsmo et al, 1997; Wang et al, 1997).

However, no statistically significant correlation could be found between p53 accumulation and p21/WAF1 expression before ADT in our study. This is in agreement with the observation that p21/WAF1 can be activated by a p53-independent pathway as well (Michieli et al, 1994). After ADT, the rate of both p53 accumulation and p21/WAF1 overexpression increased and a statistically significant positive correlation between both factors could be observed. This finding might be explained by a potentially therapy-related up-regulation of functional (wild-type) p53 protein.

Paradoxically, all p21/WAF1-positive cases in our series showed a high Ki-67 index and, occasionally, mitoses were also p21/WAF1 immunoreactive (Figure 4). Our results are in accordance with recent data from Trotter et al (1997) and from Erber et al (1998) who were able to show an aberrant p21/WAF1 expression in human cutaneous melanomas and head and-neck squamous cell carcinomas respectively. These data demonstrate that increased p21 expression is not sufficient to control growth of certain tumour entities in vivo. Moreover, Lukas et al (1997) revealed tissue-specific expression patterns of p21/WAF1 in carcinomas of the breast, ovary and endometrium which appeared to be characteristic of the tumour type. This finding suggests an abnormal function of this important cell cycle inhibitor in a subset of the tumour cells, resulting in a defective G1/S checkpoint control and leading to an excessive tumour cell proliferation in some human malignancies. Several molecular changes might cause this escape from the regular cell cycle control, including (1) mutations of the p21/WAF1 gene, (2) an alteration 'downstream' from the p53 and p21/WAF1-mediated G1/S checkpoint, or (3) changes in other factors participating in the G1/S checkpoint control (checkpoint adaptation, mutations of the CDKs or their inhibitors).

To date, direct evidence for one of these hypotheses is lacking. Nonetheless, PC was the first primary human cancer where somatic mutations of the WAF1/CIP1 gene were detected. The question of whether the mutated p21 protein is functional or non-functional is still open. The rate of 17% of WAF1 gene mutations in prostatic carcinomas reported by Gao et al (1995) is very similar to the frequency of immunohistochemical p21/WAF1 protein overexpression detected before ADT in the present study. Thus, it might be speculated that WAF1 gene mutations could lead to a mutated protein with abnormal stability. The abnormal protein – either functional or non-functional – might become detectable by immunohistochemistry in analogy to p53. Alternatively, the binding of p21/WAF1 to the cyclin/CDK complexes might be altered or inhibited by gene mutations. However, no data are as yet

© Cancer Research Campaign 1999

available concerning the correlation between WAF1 gene mutations and p21/WAF1 protein overexpression. Recently, Blagosklonny et al (1997) were able to show that the prostate cancer cell line, LNCaP, became resistant to phorbol-12-myristate-13-acetate (PMA)-induced growth arrest after transfection with the c-*myc* oncogene, despite up-regulation of p21/WAF1. Thus, continued c-*myc* expression obviously provided a circumvention of growth inhibition by tumour suppressors. Furthermore, up-regulation of cyclins might also bypass the p21-inhibitory effect by altering binding stoichiometry (Trotter et al, 1997) and p53-induced p21 WAF1 expression can mediate increased cyclin D1 synthesis itself (Chen et al, 1995).

In conclusion, our results show that the physiologic regulation of proliferation and apoptosis is defective in the majority of advanced prostatic carcinomas already before ADT. The aberrant expression of the cell cycle inhibitor p21/WAF1 in the tumour cells before androgen withdrawal characterized a subgroup of patients with unfavourable clinical course. This finding indicates that the physiologic G1/S checkpoint of the cell cycle might be impaired in these tumours. Further investigations will be necessary to elucidate the causes for this failure on DNA and RNA level. For the clinical practice, prospective investigations will have to clarify whether the immunohistochemical demonstration of p21/WAF1 overexpression in advanced prostate carcinomas can be used as a marker for hormone-refractory high-risk tumours. According to our results, these patients have no benefit from conventional androgen deprivation therapy forms.

### ACKNOWLEDGEMENTS

This paper contains parts of U Klenk's doctoral thesis. This work was supported by a grant of the Deutsche Forschungsgemeinschaft (DFG), Bonn, Germany (BA 1458/2-1) and a grant of the Curt Bohnewand Stiftung, Munich (Germany).

### REFERENCES

Ansari B, Coates PJ, Greenstein BD and Hall PA (1993) In situ end-labeling detects DNA strand breaks in apoptosis and other physiological and pathological states. *J Pathol* **170**: 1–8

Blagosklonny MV, Prabhu NS and El-Deiry WS (1997) Defects in p21/WAF1/CIP1, Rb, and c-myc signaling in phorbol ester-resistant cancer cells. *Cancer Res* **57**: 320–325

Bookstein R, MacGrogan D, Hilsenbeck SG, Sharkey F and Allred DC (1993) p53 is mutated in a subset of advanced-stage prostate cancers. *Cancer Res* **53**: 3369–3373

Breslow NE and Day NE (1980) *The Analysis of Case Control Studies*. IARC Scientific Publications, no. 32. IARC: Lyon

Bubendorf L, Sauter G, Moch H, Jordan P, Blochlinger A, Gasser TC and Mihatsch MJ (1996) Prognostic significance of Bcl-2 in clinically localized prostate cancer. *Am J Pathol* **148**: 1557–1565

Chen X, Bargonetti J and Prives C (1995) p53, through p21 (WAF1/CIP1), induces cyclin D1 synthesis. *Cancer Res* **55**: 4257–4263

Civantos F, Marcial MA, Banks ER, Ho CK, Speights VO, Drew PA, Murphy WM and Soloway MS (1995) Pathology of androgen deprivation therapy in prostate carcinoma. A comparative study of 173 patients. *Cancer* **75**: 1634–1641

Colombel M, Symmons F, Gil S, O'Toole KM, Chopin D, Benson M, Olsson CA, Korsmeyer S and Buttyan R (1993) Detection of the apoptosis-suppressing oncoprotein bcl-2 in hormone-refractory human prostate cancers. *Am J Pathol* **143**: 390–400

Eastham JA, Stapleton AMF, Gousse AE, Timme TL, Yang G, Slawin KM, Wheeler TM, Scardino PT and Thompson TC (1995) Association of *p53* mutations with metastatic prostate cancer. *Clin Cancer Res* **1**: 1111–1118

El-Deiry WS, Harper JW, O'Connor PM, Velculescu VE, Canman CE, Jackman J, Pietenpol JA, Burrell M, Hill DE, Wang Y, Wiman KG, Mercer WE, Kastan MB, Kohn KW, Elledge SJ, Kinzler KW and Vogelstein B (1994) WAF1/CIP1 is induced in p53-mediated G1 arrest and apoptosis. *Cancer Res* **54**: 1169–1174

Ellis PA, Lonning PE, Borresen-Dale A, Aas T, Geisler S, Akslen LA, Salter I, Smith IE and Dowsett M (1997) Absence of p21 expression is associated with abnormal p53 in human breast carcinomas. *Br J Cancer* **76**: 480–485

Erber R, Klein W, Andl T, Enders C, Born AI, Conradt C, Bartek J and Bosch FX (1998) Aberrant p21 (CIP1/WAF1) protein accumulation in head-and-neck cancer. *Int J Cancer* **74**: 383–389

Evan GI, Wyllie AH, Gilbert CS, Littlewood TD, Land H and Brooks M (1992) Induction of apoptosis in fibroblasts by c-myc protein. *Cell* **69**: 119–128

Gao X, Chen YQ, Wu N, Grignon DJ, Sakr W, Porter AT and Honn KV (1995) Somatic mutations of the WAF1/CIP1 gene in primary prostate cancer. *Oncogene* **11**: 1395–1398

Gavrieli Y, Sherman Y and Ben-Sasson SA (1992) Identification of programmed cell death in situ via specific labeling of nuclear DNA fragmentation. *J Cell Biol* **119**: 493–501

Gleason DF (1992) Histologic grading of prostate cancer: a perspective. *Hum Pathol* **23**: 273–279

Gleason DF (1966) Classification of prostatic carcinomas. *Cancer Chemother Rep* **50**: 125–128

Gleason DF and Mellinger GI (1974) Veterans Administration Cooperative Urological Research Group. Prediction of prognosis for prostatic adenocarcinoma by combined histologic grading and clinical staging. *J Urol* **111**: 58–64

Gomyo Y, Ikeda M, Osaki M, Tatebe S, Tsujitani S, Ikeguchi M, Kaibara N and Ito H (1997) Expression of p21 (waf1/cip1/sdi1), but not p53 protein, is a factor in the survival of patients with advanced gastric carcinoma. *Cancer* **79**: 2067–2072

Harada N, Gansauge S, Gansauge F, Gause H, Shimoyama S, Imaizumi T, Mattfeld T, Schoenberg MH and Beger HG (1997) Nuclear accumulation of p53 correlates significantly with clinical features and inversely with the expression of the cyclin-dependent kinase inhibitor p21 (WAF1/CIP1) in pancreatic cancer. *Br J Cancer* **76**: 299–305

Hollander M and Wolfe DA (1973) *Non-parametric Statistical Methods*. Wiley and Sons: New York.

Hölzel D, Klamert A and Schmidt M (1996) *Krebs*: *Häufigkeiten, Befunde und Behandlungsergebnisse*. *Perspektiven für die Krebsdiskussion und eine quantitative klinisch-epidemiologische Onkologie aus dem Tumorregister München*. W Zucker Schwerdt Verlag: Munich

Huggins C and Hodges CV (1941) Studies on prostatic cancer I. *Cancer Res* **1**: 293–297

Isaacs JT, Furuya Y and Berges R (1994) The role of androgen in the regulation of programmed cell death/apoptosis in normal and malignant prostatic tissue. *Semin Cancer Biol* **5**: 391–400

Klocker H, Culig Z, Hobisch A, Cato AC and Bartsch G (1994) Androgen receptor alterations in prostatic carcinoma. *Prostate* **25**: 266–273

Korsmeyer SJ (1992*a*) bcl-2: a repressor of lymphocyte death. *Immunol Today* **13**: 285–287

Korsmeyer SJ (1992*b*) bcl-2 initiates a new category of oncogenes: regulators of cell death. *Blood* **80**: 879–886

Krajewska M, Krajewski S, Epstein JI, Shabaik A, Sauvageot J, Song K, Kitada S and Reed JC (1996) Immunohistochemical analysis of bcl-2, bax, bcl-X, and mcl-1 expression in prostate cancers. *Am J Pathol* **148**: 1567–1576

Kubota Y, Shuin T, Uemura H, Fujinami K, Miyamoto H, Torigoe S, Dobashi Y, Kitamura H, Iwasaki Y and Danenberg K (1995) Tumor suppressor gene p53 mutations in human prostate cancer. *Prostate* **27**: 18–24

Lane DP (1992) p53, guardian of the genome. *Nature* **358**: 15–16

Levine AJ, Momand J and Finlay CA (1991) The p53 tumour suppressor gene. *Nature* **351**: 453

Li Y, Jenkins CW, Nichols MA and Xiong Y (1994) Cell cycle expression and p53 regulation of the cyclin-dependent kinase inhibitor p21. *Oncogene* **9**: 2261–2268

Lukas J, Groshen S, Saffari B, Niu N, Reles A, Wen WH, Felix J, Jones LA, Hall FL and Press MF (1997) WAF1/Cip1 gene polymorphism and expression in carcinomas of the breast, ovary, and endometrium. *Am J Pathol* **150**: 167–175

Maelandsmo GM, Holm R, Fodstad O, Kerbel RS and Florenes VA (1997) Cyclin kinase inhibitor p21/WAF1/CIP1 in malignant melanoma. Reduced expression in metastatic lesions. *Am J Pathol* **149**: 1813–1822

McDonnell TJ, Troncoso P, Brisbay SM, Logothetis C, Chung LWK, Hsieh J-R, Tu S-M and Campbell ML (1992) Expression of the protooncogene bcl-2 in the prostate and its association with emergence of androgen-independent prostate cancer. *Cancer Res* **52**: 6940–6944

Michieli P, Chedid M, Lin D, Pierce JH, Mercer WE and Givol D (1994) Induction of WAF1/CIP1 by a p53-independent pathway. *Cancer Res* **54**: 3391–3395

 © Cancer Research Campaign 1999

Miyamoto KK, McSherry SA, Dent GA, Sar M, Wilson EM, French FS, Sharief Y and Mohler JL (1993) Immunohistochemistry of the androgen receptor in human benign and malignant prostate tissue. *J Urol* **149**: 1015–1019

Navone NM, Troncoso P, Pisters LL, Goodrow TL, Palmer JL, Nichols WW, von Eschenbach AC and Conti CJ (1993) p53 protein accumulation and gene mutation in the progression of human prostate carcinoma. *J Natl Cancer Inst* **85**: 1657–1669

Peterziel H, Culig Z, Stober J, Hobisch A, Radmayr C, Bartsch G, Klocker H and Cato AC (1995) Mutant androgen receptors in prostatic tumors distinguish between amino-acid-sequence requirements for transactivation and ligand binding. *Int J Cancer* **63**: 544–550

Piffko J, Bankfalvi A, Öfner D, Joos U, Böcker W and Schmid KW (1995) Immunohistochemical detection of p53 protein in archival tissues from squamous cell carcinomas of the oral cavity using wet autoclave antigen retrieval. *J Pathol* **176**: 69–75

Polyak K, Xia Y, Zweier JL, Kinzler KW and Vogelstein B (1997) A model for p53-induced apoptosis. *Nature* **389**: 300–305

Shurbaji MS, Kalbfleisch JH and Thurmond TS (1995) Immunohistochemical detection of p53 protein as a prognostic indicator in prostate cancer. *Hum Pathol* **26**: 106–109

Stewart BW (1994) Mechanisms of apoptosis: Intergration of genetic, biochemical, and cellular indicators. *J Natl Cancer Inst* **86**: 1286–1296

Suzuki H, Akakura K, Komiya A, Aida S, Akimoto S and Shimazaki J (1996) Codon 877 mutation in the androgen receptor gene in advanced prostate cancer: relation to antiandrogen withdrawal syndrome. *Prostate* **29**: 153–158

Taplin ME, Bubley GJ, Shuster TD, Frantz ME, Spooner AE, Ogata GK, Keer HN and Balk SP (1995) Mutation of the androgen-receptor gene in metastatic androgen-independent prostate cancer. *N Engl J Med* **332**: 1393–1398

Trapman J and Brinkmann AO (1996) The androgen receptor in prostate cancer. *Pathol Res Pract* **192**: 752–760

Trotter MJ, Tang L and Tron VA (1997) Overexpression of the cyclin-dependent kinase inhibitor p21 (WAF1/CIP1) in human cutaneous malignant melanoma. *J Cutan Pathol* **24**: 265–271

Visakorpi T, Kallioniemi OP, Heikkinen A, Koivula T and Isola J (1992) Small subgroup of aggressive, highly proliferative prostatic carcinomas defined by p53 accumulation. *J Natl Cancer Inst* **84**: 883–887

Visakorpi T, Hyytinen E, Koivisto P, Tanner M, Keinanen R, Palmberg C, Palotie A, Tammela T, Isola J and Kallioniemi OP (1995) In vivo amplification of the androgen receptor gene and progression of human prostate cancer. *Nat Genet* **9**: 401–406

Wang A, Yoshimi N, Tanaka T and Mori H. (1997) WAF1 expression and p53 mutations in human colorectal cancers. *J Cancer Res Clin Oncol* **123**: 118–123

Wertz IE, Deitch AD, Gumerlock PH, Gandour Edwards R, Chi SG and de Vere White RW (1996) Correlation of genetic and immunodetection of TP53 mutations in malignant and benign prostate tissues. *Hum Pathol* **27**: 573–580

Westin P, Stattin P, Damber JE and Bergh A (1995) Castration therapy rapidly induces apoptosis in a minority and decreases cell proliferation in a majority of human prostatic tumors. *Am J Pathol* **146**: 1368–1375

*© Cancer Research Campaign 1999*

## References

[PDF_00758] Ansari B., Coates P. J., Greenstein B. D., Hall P. A. (1993). In situ end-labelling detects DNA strand breaks in apoptosis and other physiological and pathological states.. J Pathol.

[PDF_00761] Blagosklonny M. V., Prabhu N. S., El-Deiry W. S. (1997). Defects in p21WAF1/CIP1, Rb, and c-myc signaling in phorbol ester-resistant cancer cells.. Cancer Res.

[PDF_00764] Bookstein R., MacGrogan D., Hilsenbeck S. G., Sharkey F., Allred D. C. (1993). p53 is mutated in a subset of advanced-stage prostate cancers.. Cancer Res.

[PDF_00769] Bubendorf L., Sauter G., Moch H., Jordan P., Blöchlinger A., Gasser T. C., Mihatsch M. J. (1996). Prognostic significance of Bcl-2 in clinically localized prostate cancer.. Am J Pathol.

[PDF_00772] Chen X., Bargonetti J., Prives C. (1995). p53, through p21 (WAF1/CIP1), induces cyclin D1 synthesis.. Cancer Res.

[PDF_00774] Civantos F., Marcial M. A., Banks E. R., Ho C. K., Speights V. O., Drew P. A., Murphy W. M., Soloway M. S. (1995). Pathology of androgen deprivation therapy in prostate carcinoma. A comparative study of 173 patients.. Cancer.

[PDF_00777] Colombel M., Symmans F., Gil S., O'Toole K. M., Chopin D., Benson M., Olsson C. A., Korsmeyer S., Buttyan R. (1993). Detection of the apoptosis-suppressing oncoprotein bc1-2 in hormone-refractory human prostate cancers.. Am J Pathol.

[PDF_00781] Eastham J. A., Stapleton A. M., Gousse A. E., Timme T. L., Yang G., Slawin K. M., Wheeler T. M., Scardino P. T., Thompson T. C. (1995). Association of p53 mutations with metastatic prostate cancer.. Clin Cancer Res.

[PDF_00788] Ellis P. A., Lonning P. E., Borresen-Dale A., Aas T., Geisler S., Akslen L. A., Salter I., Smith I. E., Dowsett M. (1997). Absence of p21 expression is associated with abnormal p53 in human breast carcinomas.. Br J Cancer.

[PDF_00791] Erber R., Klein W., Andl T., Enders C., Born A. I., Conradt C., Bartek J., Bosch F. X. (1997). Aberrant p21(CIP1/WAF1) protein accumulation in head-and-neck cancer.. Int J Cancer.

[PDF_00794] Evan G. I., Wyllie A. H., Gilbert C. S., Littlewood T. D., Land H., Brooks M., Waters C. M., Penn L. Z., Hancock D. C. (1992). Induction of apoptosis in fibroblasts by c-myc protein.. Cell.

[PDF_00796] Gao X., Chen Y. Q., Wu N., Grignon D. J., Sakr W., Porter A. T., Honn K. V. (1995). Somatic mutations of the WAF1/CIP1 gene in primary prostate cancer.. Oncogene.

[PDF_00799] Gavrieli Y., Sherman Y., Ben-Sasson S. A. (1992). Identification of programmed cell death in situ via specific labeling of nuclear DNA fragmentation.. J Cell Biol.

[PDF_00804] Gleason D. F. (1966). Classification of prostatic carcinomas.. Cancer Chemother Rep.

[PDF_00802] Gleason D. F. (1992). Histologic grading of prostate cancer: a perspective.. Hum Pathol.

[PDF_00806] Gleason D. F., Mellinger G. T. (1974). Prediction of prognosis for prostatic adenocarcinoma by combined histological grading and clinical staging.. J Urol.

[PDF_00810] Gomyo Y., Ikeda M., Osaki M., Tatebe S., Tsujitani S., Ikeguchi M., Kaibara N., Ito H. (1997). Expression of p21 (waf1/cip1/sdi1), but not p53 protein, is a factor in the survival of patients with advanced gastric carcinoma.. Cancer.

[PDF_00814] Harada N., Gansauge S., Gansauge F., Gause H., Shimoyama S., Imaizumi T., Mattfeld T., Schoenberg M. H., Beger H. G. (1997). Nuclear accumulation of p53 correlates significantly with clinical features and inversely with the expression of the cyclin-dependent kinase inhibitor p21(WAF1/CIP1) in pancreatic cancer.. Br J Cancer.

[PDF_00827] Isaacs J. T., Furuya Y., Berges R. (1994). The role of androgen in the regulation of programmed cell death/apoptosis in normal and malignant prostatic tissue.. Semin Cancer Biol.

[PDF_00830] Klocker H., Culig Z., Hobisch A., Cato A. C., Bartsch G. (1994). Androgen receptor alterations in prostatic carcinoma.. Prostate.

[PDF_00834] Korsmeyer S. J. (1992). Bcl-2 initiates a new category of oncogenes: regulators of cell death.. Blood.

[PDF_00832] Korsmeyer S. J. (1992). Bcl-2: a repressor of lymphocyte death.. Immunol Today.

[PDF_00836] Krajewska M., Krajewski S., Epstein J. I., Shabaik A., Sauvageot J., Song K., Kitada S., Reed J. C. (1996). Immunohistochemical analysis of bcl-2, bax, bcl-X, and mcl-1 expression in prostate cancers.. Am J Pathol.

[PDF_00839] Kubota Y., Shuin T., Uemura H., Fujinami K., Miyamoto H., Torigoe S., Dobashi Y., Kitamura H., Iwasaki Y., Danenberg K. (1995). Tumor suppressor gene p53 mutations in human prostate cancer.. Prostate.

[PDF_00842] Lane D. P. (1992). Cancer. p53, guardian of the genome.. Nature.

[PDF_00843] Levine A. J., Momand J., Finlay C. A. (1991). The p53 tumour suppressor gene.. Nature.

[PDF_00845] Li Y., Jenkins C. W., Nichols M. A., Xiong Y. (1994). Cell cycle expression and p53 regulation of the cyclin-dependent kinase inhibitor p21.. Oncogene.

[PDF_00848] Lukas J., Groshen S., Saffari B., Niu N., Reles A., Wen W. H., Felix J., Jones L. A., Hall F. L., Press M. F. (1997). WAF1/Cip1 gene polymorphism and expression in carcinomas of the breast, ovary, and endometrium.. Am J Pathol.

[PDF_00851] Maelandsmo G. M., Holm R., Fodstad O., Kerbel R. S., Flørenes V. A. (1996). Cyclin kinase inhibitor p21WAF1/CIP1 in malignant melanoma: reduced expression in metastatic lesions.. Am J Pathol.

[PDF_00854] McDonnell T. J., Troncoso P., Brisbay S. M., Logothetis C., Chung L. W., Hsieh J. T., Tu S. M., Campbell M. L. (1992). Expression of the protooncogene bcl-2 in the prostate and its association with emergence of androgen-independent prostate cancer.. Cancer Res.

[PDF_00858] Michieli P., Chedid M., Lin D., Pierce J. H., Mercer W. E., Givol D. (1994). Induction of WAF1/CIP1 by a p53-independent pathway.. Cancer Res.

[PDF_00864] Miyamoto K. K., McSherry S. A., Dent G. A., Sar M., Wilson E. M., French F. S., Sharief Y., Mohler J. L. (1993). Immunohistochemistry of the androgen receptor in human benign and malignant prostate tissue.. J Urol.

[PDF_00867] Navone N. M., Troncoso P., Pisters L. L., Goodrow T. L., Palmer J. L., Nichols W. W., von Eschenbach A. C., Conti C. J. (1993). p53 protein accumulation and gene mutation in the progression of human prostate carcinoma.. J Natl Cancer Inst.

[PDF_00871] Peterziel H., Culig Z., Stober J., Hobisch A., Radmayr C., Bartsch G., Klocker H., Cato A. C. (1995). Mutant androgen receptors in prostatic tumors distinguish between amino-acid-sequence requirements for transactivation and ligand binding.. Int J Cancer.

[PDF_00875] Piffko J., Bankfalvi A., Ofner D., Joos U., Böcker W., Schmid K. W. (1995). Immunohistochemical detection of p53 protein in archival tissues from squamous cell carcinomas of the oral cavity using wet autoclave antigen retrieval.. J Pathol.

[PDF_00879] Polyak K., Xia Y., Zweier J. L., Kinzler K. W., Vogelstein B. (1997). A model for p53-induced apoptosis.. Nature.

[PDF_00881] Shurbaji M. S., Kalbfleisch J. H., Thurmond T. S. (1995). Immunohistochemical detection of p53 protein as a prognostic indicator in prostate cancer.. Hum Pathol.

[PDF_00884] Stewart B. W. (1994). Mechanisms of apoptosis: integration of genetic, biochemical, and cellular indicators.. J Natl Cancer Inst.

[PDF_00886] Suzuki H., Akakura K., Komiya A., Aida S., Akimoto S., Shimazaki J. (1996). Codon 877 mutation in the androgen receptor gene in advanced prostate cancer: relation to antiandrogen withdrawal syndrome.. Prostate.

[PDF_00889] Taplin M. E., Bubley G. J., Shuster T. D., Frantz M. E., Spooner A. E., Ogata G. K., Keer H. N., Balk S. P. (1995). Mutation of the androgen-receptor gene in metastatic androgen-independent prostate cancer.. N Engl J Med.

[PDF_00892] Trapman J., Brinkmann A. O. (1996). The androgen receptor in prostate cancer.. Pathol Res Pract.

[PDF_00894] Trotter M. J., Tang L., Tron V. A. (1997). Overexpression of the cyclin-dependent kinase inhibitor p21(WAF1/CIP1) in human cutaneous malignant melanoma.. J Cutan Pathol.

[PDF_00900] Visakorpi T., Hyytinen E., Koivisto P., Tanner M., Keinänen R., Palmberg C., Palotie A., Tammela T., Isola J., Kallioniemi O. P. (1995). In vivo amplification of the androgen receptor gene and progression of human prostate cancer.. Nat Genet.

[PDF_00897] Visakorpi T., Kallioniemi O. P., Heikkinen A., Koivula T., Isola J. (1992). Small subgroup of aggressive, highly proliferative prostatic carcinomas defined by p53 accumulation.. J Natl Cancer Inst.

[PDF_00904] Wang A., Yoshimi N., Ino N., Tanaka T., Mori H. (1997). WAF1 expression and p53 mutations in human colorectal cancers.. J Cancer Res Clin Oncol.

[PDF_00906] Wertz I. E., Deitch A. D., Gumerlock P. H., Gandour-Edwards R., Chi S. G., de Vere White R. W. (1996). Correlation of genetic and immunodetection of TP53 mutations in malignant and benign prostate tissues.. Hum Pathol.

[PDF_00909] Westin P., Stattin P., Damber J. E., Bergh A. (1995). Castration therapy rapidly induces apoptosis in a minority and decreases cell proliferation in a majority of human prostatic tumors.. Am J Pathol.

[PDF_00784] el-Deiry W. S., Harper J. W., O'Connor P. M., Velculescu V. E., Canman C. E., Jackman J., Pietenpol J. A., Burrell M., Hill D. E., Wang Y. (1994). WAF1/CIP1 is induced in p53-mediated G1 arrest and apoptosis.. Cancer Res.

